# Parental Knowledge and Perceptions on Prevention of Sudden Unexpected Infant Death and Infant Care

**DOI:** 10.3390/children10091550

**Published:** 2023-09-14

**Authors:** Dziyana Nazaruk, Ana M. Palacios, Joanne Chopak-Foss, Tilicia L. Mayo-Gamble, Nandi A. Marshall

**Affiliations:** Department of Health Policy and Community Health, Jiann-Ping Hsu College of Public Health, Georgia Sothern University, 11935 Abercorn Street, Savannah, GA 31419, USA

**Keywords:** infant care, parental knowledge, parental perceptions, infant health, prevention of sudden unexpected infant death, SUID

## Abstract

(1) Background: The study’s purpose was to explore the knowledge, perceptions, and confidence of mothers about infant care to reduce the risk of sudden unexpected infant death. (2) Methods: A purposeful sampling method was used to recruit 15 first-time mothers from Georgia with infants under 1 year of age. The researchers utilized the Socio-ecological model to report the results. Participants also provided recommendations on how to improve infant care and reduce the risk of SUID. (3) Results: The confidence level of infant care among most participants was low but increased over time. Mothers’ knowledge level about the prevention of SUID was high, but poor emotional health could hurt their parental abilities. Most participants recognized medical providers as the main source of reliable information. However, a lack of emotional and physical support was reported by mothers. (4) Conclusions: Results suggested that a more holistic approach to infant care is needed. The healthcare system and communities should provide more physical, social, and mental support to first-time mothers, a consolidated approach to care before and after birth, and easy access to services at all stages of the process to reduce the risk of SUID.

## 1. Introduction

Infant mortality rate (IMR) is used as an overall indicator of a nation’s health. IMR is defined as the ratio of infant deaths to live births in a given year [1]. According to America’s Health Ranking Annual Report in 2018, the US infant mortality rate was 5.9 deaths per 1000 live infant births, which placed the US at No. 33 out of 36 countries with the world’s largest economy in 2018 [2]. Indeed, progress has been made to reduce the infant mortality rate over the past decade, but the pace of change is occurring slower in the USA than in other comparable countries [3].

Significant disparities in infant mortality exist among different states. Georgia’s IMR in 2018 was 7.6 deaths per 1000 live infant births, placing Georgia at No.45 out of 50 states. Furthermore, infant mortality in Georgia has been on the rise since 2014, when it was the lowest at 6.5 deaths per 1000 live infant births [2].

Beginning in the 1990, the United States had a sharp decline in sudden unexpected/unexplained causes of death (SUID); however, the decrease has slowed down considerably since 1999 [4]. In 2017, the top ten leading causes of infant death were responsible for 67.8% of all infant deaths in the United States. SUID and death from unintentional injuries were in the third and fourth place. Furthermore, death from unintentional injuries increased by 10.7% from 2016 to 2017 [1]. Not only are unintentional injuries, such as falls, burns and drowning, some of the leading causes of infant mortality, but they are also some of the major causes of morbidity among children.

Therefore, what has been carried out to date to decrease SUID? The CDC’s Division of Reproductive Health (DRH) provides scientific leadership in SUID by sharing up-to-date information about SUID rates and circumstances linked with SUID and has SUID monitoring programs in twenty-two states and jurisdictions, which cover one in three SUID cases. The SAFE TO SLEEP campaign focuses on spreading safe sleep strategies and educational materials to reduce the risk of SUID and other sleep-related deaths. The “Protect the Ones You Love” Initiative was launched by CDC to raise parents’ awareness about the leading causes of child injury. “The 1000 grandmothers’ project” was an education project that focused on tribal elders to educate young native parents on sleep practices for infants [5]. However, all these programs reach a specific segment of the population or have a narrow focus, addressing only one cause of SUID. Additionally, a systematic review and meta-analysis that included 10 randomized control trials suggested that the integration of universal interventions to enhance first-time parents’ self-efficacy could support successful transition into parenthood [6].

In order to address this outcome more holistically, this study sought to explore the parental knowledge, perceptions, and confidence of mothers about the prevention of SUID and other aspects of infant care. Current study exclusively refers to the term “parental” to describe those who gave birth to the infant.

## 2. Materials and Methods

### 2.1. Design and Approach

In this qualitative research, the transferability (vs. external validity), credibility, and dependability (vs. reliability) of the study were established [7] (234). The following research question and sub-aims guided the study: What is the knowledge, perceptions, confidence, and social support of first-time mothers with children under 1 year of age on the prevention of SUID and infant care? Research sub-questions: (1) How do participants describe their knowledge, perception, and confidence in the prevention of SUID and infant care? (2) What role does social support play in the prevention of SUID and infant care? (3) What role does community support play in the prevention of SUID and infant care? The current study utilized a Socio-ecological model to design the semi-structured interview guide and report the findings. This analytical framework was developed by sociologists in the 1970s. The framework’s focus is on behavior influences at the individual, interpersonal, and community levels as well as the interaction between these levels. The strength of this approach is that it helps to develop short- and long-term solutions on multiple levels [8].

### 2.2. Participant Recruitment

The investigators conducted a literature review to identify the best strategy to reach the participants. Based on the results, the recruitment strategy utilized social media channels to recruit participants from various geographic areas in Georgia. Specifically, “moms” groups on Instagram and Facebook channels were used to recruit the participants. The PI contacted the managers of these social media groups to get permission to distribute the study’s flyer. The flyers contained information about the research project, contact information, and link to the survey in Qualtrics, (Qualtrics, 2022, Provo, UT, USA) where parents could register for the study.

All the survey responses were screened for eligibility, and participants were contacted by their preferred method to schedule an in-depth interview and provide a consent form. After the participants provided signed informed consent and eligibility, the researchers scheduled in-depth interviews. The provided contact information was securely stored and destroyed after the completion of the study. Inclusion criteria for the participants included first-time mothers with children under 1 year of age, female, Georgia residents, over 18 years of age, and fluent in English. Exclusion criteria included pregnant women, not first-time mothers, and women with children over 1 year of age.

Prior to the interview, the researcher explained the purpose of the study, the benefits and risks of the study, and ensured confidentiality. It was made clear to participants that participation in the study was voluntary and that they may withdraw at any time. The consent form also contained the researcher’s contact information for any participant who had a question. Participants signed the informed consent document electronically and emailed it back to the investigators before the interview.

### 2.3. Instrumentation/Data Collection and Analysis

A semi-structured question guide was utilized for in-depth interviews to obtain qualitative information, and all interviews were conducted over the phone. The interview guide was based on research queries and the ideological concepts of the Socio-ecological model (Appendix A). For the qualitative data examination, researchers performed the thematic analysis. The in-depth interviews were recorded over the phone. All the interviews were transcribed verbatim and coded by using the qualitative software NVivo 11. The researchers first read each transcript and made notes. Then, the researchers aggregated all words and phrases from all the interviews. Once the list was created, the researchers looked for overlapping or similar categories. Utilizing a Socio-ecological model, these categories were further refined and reduced. Finally, all the data were entered in a Microsoft Word document, and the findings were presented from this file.

## 3. Results

A total of 15 participants were interviewed. Table 1 shows the sociodemographic characteristics of the participants from Georgia who participated in this study (Table 1).

### 3.1. Individual Level

The first part of the interview addressed the first research sub-question. This section also explores the main source of information on infant care and prevention of SUID for first-time mothers. Finally, it addresses mothers’ concerns and feelings about giving birth and providing care for their infants.

Theme: “Low confidence about infant care at the beginning but increases over time”.

All participants were asked to describe their level of confidence when taking care of an infant. Many of the participants described their experience as overwhelming at first. However, as time progressed, first-time mothers felt more confident to take care of their children: “At first, I was scared when I left the hospital…we got home and we’re like okay, I guess this is what we are going to do…after the first couple of weeks I felt more confident but when I first left, I was just like afraid in general as far as the wellbeing of my child just because it’s my first time I didn’t really know what to expect”?

Theme: “Breastfeeding, safe sleep, car seats, CPR training, and being intuitive are essential for first time mothers”.

Participants were asked what they thought was essential when taking care of their infant. The participants perceived breastfeeding, proper car seat use, and safe sleep practices as essential: “So that’s a first, obviously anything to do with safety, in terms of sleeping arrangements for the baby, you know”.

Another participant said: “Um safety is one of the biggest things, making sure the car seat is installed properly and um basically obviously making sure the child is safe is number one priority”.

Theme: “Breastfeeding was important, but many women felt pressure to breastfeed”.

Most women felt that breastfeeding was essential for the mothers’ and infants’ health. Also, they expressed that breastfeeding provided a chance to bond and protect their child, but many mothers felt pressure to breastfeed, and if they were not successful, many felt guilty: “While it was a little bit of pressure, which I didn’t appreciate. They were also pro-breastfeeding hospitals, which I love”.

Another mother stated: “So, my breastfeeding at the beginning was not great. Um… He ended up having jaundice. And I kind of blame it on myself because I felt like he wasn’t getting any breast milk”.

Theme: “Emotional journey and feeling of loneliness after giving birth”.

Even though there was no specific question that addressed the emotional state of mothers during the interview, many participants felt very emotional, anxious, sleep deprived, and lonely after giving birth: “Becoming parents for the first time, the whole emotional journey and changes, especially for me, as you know, my body’s biological changes, and then my emotional changes that I go through. I guess that’s why they call it the fourth trimester, when you have a baby…and so I think that’s also part of infant care. Not forgetting about yourself, not forgetting about the dad”.

### 3.2. Interpersonal Level

The second part of the interview addressed the second research sub-question. It focused on the interpersonal level of the Socio-ecological model, which refers to social influences from friends and family on the infant care of first-time mothers.

Theme: “Mothers are the infant’s primary caregivers, but partial help comes from family and friends”.

We asked participants how much physical assistance they received when taking care of their infant, and the majority stated that they mostly were providing care alone. However, many identified their family, spouses, and friends as a partial support for infant care: “So, I live like three and a half hours away from my family so that’s difficult. I mean, they will come to visit but they have their own life as well”.

Another participant stated: “His dad was there. He was helpful…he did work a normal 8 to 5. My mom was there. She was in town maybe three or four times a month and was helpful in the first month”.

Theme: “Information provided by family is not evidence-based and “old school”.

Most participants felt that the received information about infant care from family was not reliable, because it was outdated and not based on recent research studies: “I was raised, my parents back in the 80s raised me with all sorts of bumpers in the cribs and pillows and (Laughs) toys”.

Participants talked to a great extent about how infant care information changes rapidly and cited that as the main reason not to trust their parents’ and grandparents’ infant care advice: “Like, my grandma came to visit him. And she put a blanket in his bassinet, which obviously like, take it out…a lot of pressure to not offend anyone. Having older, older people come in and give their advice”.

Theme: “First-time parents perceive emotional and physical family and friends support for infant care as important”.

As previously mentioned, most women did not trust the infant care information provided by their family because it was not scientifically backed. However, they expressed that when it came to emotional and physical support, family and friends played an essential role in their well-being: “it’s still, you know, phone calls away. Uh video, FaceTime that was nice. And to have support and love, like I said, was important for me. And for my emotional well-being”.

Another mother said: “It’s so nice to have people I can trust to have him when I can’t be with him…so, it’s important to have some sort of support whether it’s family or friends”.

Theme: “Virtual or in-person mom’s groups are a great source of social support for new moms”.

In addition to family and friends, many participants talked about the importance of social interactions with other parents. They wanted to connect with someone who went through something similar or was currently going through the same experiences as they were. They also relied on social “mom’s groups” when they had various questions about infant care: “Like more newly moms that you want to meet… more how to find people who the same age and is going through the same thing. That would be nice”.

### 3.3. Community Level

The next part of the interview addressed the third research sub-question. This part focused on the community level of the Socio-ecological model, where questions addressed the role of social relationships and received information at that level.

Theme: “Medical information in infant care was essential when compared to family, friends, and other sources”.

When asked what the essential source of information was when it came to infant care, participants stated that they trusted the information provided by medical professionals the most because it was research-based and up to date: “Things that a doctor would know that’s different that a mother raising a child 10 years ago wouldn’t know”.

Most participants said that the information provided by the medical professionals was easy to understand. They also felt comfortable asking questions in medical settings. Women described the medical language on infant care as helpful, clear, and easy to understand.

Theme: “Participants wanted to meet the pediatrician before giving birth, because they were considered? the essential source of infant care information”.

Mothers mentioned that if they had any major concerns, they would contact the pediatrician first. They felt that they could trust their pediatrician the most compared to the hospital, family, and OBGYN: “Pediatrician knows more than anybody else…could really provide information, especially when it comes to medicines or something like that, or anything to do with my child”.

Participants met the pediatrician after giving birth to their child. However, most of them wanted to meet the pediatrician before giving birth to be better prepared to take care of their infant: “I asked about that. I was kind of wanting to meet them…and the receptionist was like “No, you know once you have a baby you can come in”.

Theme: “The information and support provided by the Obstetrics and Gynecology physician (OBGYN) focused mostly on the female parent’s health before giving birth but was lacking after birth”.

Most women described that their OBGYN support was very important during pregnancy and focused mostly on the mother’s physical health. Some OBGYNs provide information about breastfeeding and antenatal classes. However, women did not receive information about infant care: “But maybe a little more to check in and not like a follow up like hey how are you doing that’s great bye, but actually be like how you are feeling”?

Theme: “Printed infant care information provided by the hospital was overwhelming, but personal conversations were very helpful”.

Most of the participants gave birth at the hospital. All of them received a printed packet of flyers on infant care, some watched hospital videos, and some had one-on-one conversations about infant care prior to discharge. Many felt that the packet and provided information were quite overwhelming because it happened right after giving birth: “I was just super overwhelmed. It was a lot in the brain all at once”.

However, participants found it helpful when hospital staff would take their time and had conversations about infant care, or even provided demonstrations: “When it came to informing me on things, I had questions about like him getting shots or you know, stuff like that… they were very informative and if I didn’t understand they would take the time to explain it to me. I found it very helpful”.

In conclusion, mothers trusted medical information when it came to specific infant care questions. For all the other infant questions, women relied on intuition and the internet, specifically “mom’s groups”, podcasts, google, and phone applications.

### 3.4. Recommendations

When asked what some major barriers are to accessing the infant care information, participants explained that even though they would like to attend more antenatal classes and workshops, they struggled with finding classes that were free of charge or have available spots: “I was taking those classes…they were like 75 dollars here 50 dollars here 40 dollars here that adds up”?

Some women also talked about having more online class options, due to the lack of transportation, busy schedule, and COVID concerns: “Of course, COVID…I didn’t feel comfortable taking her anywhere near it”.

When we asked participants to provide recommendations, three categories emerged: resources, interpersonal communication, and delivery of services. When talking about additional resources, mothers expressed that they would like to receive more information before giving birth on: (1) List of products/supplies, (2) link to resources, (3) more video tutorials, (4) information on car seats and breastfeeding, and (5) more classes that are affordable and easy to access.

Women talked about the importance of interpersonal communication and social support. Some of the suggestions that they have provided were: (1) More verbal communication, (2) more personal interactions with the medical professionals, and (3) more virtual and in-person mom groups. Women suggested the following to improve the delivery of services: (1) More programs and/or services to support mothers after birth that are free of charge, (2) more follow-up after birth to check on mothers’ mental health, not only physical health, and (3) more information and interactions with the pediatrician before giving birth.

Finally, most participants talked about the importance of technology as a method to deliver information on infant care. Many utilized Facebook, Instagram, and podcasts when they had any general questions about infant care and suggested creating a phone application that would be a “one-stop” for the most updated information on infant care, interactions with medical professionals, social connection with other mothers, and mental health services support for mothers. They have mentioned currently using applications that have small charges or free of charge but did not encompass all the aspects of being a new parent.

## 4. Discussion

The current study explored the individual-level factors associated with SUID prevention and infant care. Most mothers had a low confidence level when caring for their infants upon release from the hospital, but it increased over time. There is a lack of research that focuses on mothers’ confidence and infant care. However, previous findings showed an association between maternal confidence and parenting stress [9].

According to the most recent recommendations, the main protective factors of SUID are safe sleep practices, breastfeeding, and healthy behaviors during pregnancy [10], which most of our participants were knowledgeable about. However, the emotional journey and feeling of loneliness that women experienced after pregnancy could affect the ability to successfully perform their parental duties. According to previous research, infants of mothers with postnatal depressive symptoms or depression had an increased risk of hospitalization and death in the first year of life [11]. Additionally, when women were asked about breastfeeding, they all believed it was very important for the health of the infant and a great bonding experience. However, most women blamed themselves for challenges associated with breastfeeding and felt judged by other people including medical personnel. This finding is consistent with the literature, even though most challenges associated with breastfeeding are not personal but structural [12].

The current study observed that mothers were the infant’s primary caregiver. However, family and partners provided partial support, especially during the early stages of infancy. The most valued support was emotional and physical. Previous research has observed that care provided by the relative could be beneficial and positively influences infant communication and interpersonal skills [13].

The participants also expressed the importance of social connections with mothers that were going or already went through similar experiences, which is consistent with the literature [14]. There was some mistrust of the information provided by family and friends because it was not scientifically based and likely obsolete. Women perceived medical providers, specifically the pediatrician, as the main source of reliable information on infant care.

The current study also analyzed community-level support for first-time mothers. Our findings suggest that women received a large amount of information and support during pregnancy from their OBGYN but were lacking medical support after giving birth. Even though participants were satisfied with the information and care provided by the pediatrician, they expressed the need to meet the pediatrician before giving birth to be better prepared for infant care. Previous studies established the importance of expecting mothers’ thoughts and feelings about their unborn infant and future interactions with their child [15]. Finally, the information provided by the hospital was quite overwhelming due to being stressed during the first days after delivery.

Some of the recommendations provided by women to be better prepared for infant care were additional resources, interpersonal communication, and the delivery of services. The preferred communication channel for the delivery of infant care information was a phone application that would contain up-to-date information, provide access to healthcare providers, and provide social interactions with other mothers. This is supported by a randomized controlled trial that observed a positive effect of mobile-based parental education programs in prevention of early childhood injuries. In addition, parents found the use of the mobile program convenient [16].

Some of the limitations of the study include the following: most participants were from urban areas in Georgia, and mostly White women living with a partner. This is important to note because the risk of SUID might depend on multiple sociodemographic factors, and also our findings may have a limited external validity. Another possible bias was the utilization of an online recruiting strategy, which could limit generalizability. Despite the limitations, we believe that the results of the current report make an important contribution to literature on supporting mothers in caring for their infants.

Current findings identified the factors and needs of mothers on each level of the Socio-ecological model. Further, findings demonstrated interconnectivity between all levels of the model. Women expressed that the provided medical services are compartmentalized. There is a large amount of attention on women during pregnancy but not nearly enough after giving birth. There is strong support for infant care provided by a pediatrician after birth, but a lack of preparation for infant care during pregnancy. For mothers to feel more competent to offer high-quality care for their infants, they need to have more emotional, social, and physical support from family, friends, and society, a more integrated approach to care before and after birth, and easy access to services and technology at all stages of the process. Finally, we hope that the results of this study will help health professionals and community organizations determine the information and support that needs to be provided for new mothers.

## Figures and Tables

**Table 1:** Demographic characteristics of the participants, N = 15.

Characteristic	N (%)
**Sex, Female**	
Age	
18–28	6 (40.0)
29–39	6 (40.0)
40–49	3 (20.0)
Total	15 (100.0)
**Race**	
White, Non-Hispanic	11 (73.3)
Black/African American	3 (20.0)
Hispanic	1 (6.0)
Total	15 (100.0)
**Marital Status**	
Married	9 (60.0)
Cohabiting	4 (26.7)
Single	2 (13.3)
Total	15 (100.0)

## Data Availability

Data is unavailable due to privacy.

## Appendix A

Semi-structured interview guide (Guided by the Socio-ecological model):

Individual level:

1. What do you know about infant care? Where did you learn this information from?
2. What do you believe is essential when taking care of the infant?
3. What safe practices did you implement when taking care of an infant?
4. How confident do you feel to take care of the infant?

Family/Peer level:

1. What kind of information did you receive from your family or friends about infant care?
2. How important is your family support when taking care of the infant?
3. Does anyone help you to take care of your child?

Community level:

1. What type of information have you received from your OBGYN about infant care?
2. What kind of language did your healthcare provider use? How clear was the provided information?
3. What type of information have you received in the hospital about infant care when delivering the baby? How clear was the provided information?
4. How important is your OBGYN support? Compared to your friends and family support?
5. What additional information would you like to receive from your OBGYN or hospital staff?
6. Can you think of any ways that your OBGYN could improve communication with you?
7. Have you utilized any other services/educational workshops/classes in your community about infant care? If yes, which ones? How satisfied were you with the provided information?

Structural:

1. Are there any barriers to accessing the information/services on infant care? Probe: distance, time, resources, availability of services, confidentiality of information.
2. What are your recommendations on improving the existing services? Providing new ones?
3. How would you like to receive the information on infant care?

## References

[1] Murphy S.L., Xu J.Q., Kochanek K.D., Arias E. (2018). Mortality in the United States, 2017.

[2] (2022). America’s Health Rankings Analysis of CDC WONDER Online Database, Linked Birth/Infant Death Files, United Health Foundation. https://www.americashealthrankings.org/.

[3] Kamal R., Hudman J., McDermott D. (2019). What Do We Know about Infant Mortality in the US and Comparable Countries? Peterson-KFF Health System Tracker. https://www.healthsystemtracker.org/chart-collection/infant-mortality-u-s-compare-countries/.

[4] Center for Disease Control and Prevention (2019). Trends in Sudden Unexpected Infant Death by Cause, 1990–2018. CDC/NCHS, National Vital Statistics System, Mortality Files.

[5] Center for Disease Control and Prevention (2020). Learn What CDC Is Doing about Sudden Unexpected Infant Death (SUID) Learn What CDC Is Doing about Sudden Unexpected Infant Death (SUID)|CDC.

[6] Nur A., Liyana A., Wilson T., Shefaly S. (2018). Enhancing first-time parents’ self-efficacy: A systematic review and meta-analysis of universal parent education interventions’ efficacy. Int. J. Nurs. Stud..

[7] Patton M. (2002). Qualitative Research & Evaluation Methods.

[8] DiClemente R.J., Salazar L., Crosby R.A. (2019). Health Behavior Theory for Public Health.

[9] Liu C.-C., Chen Y.-C., Yeh Y.-P., Hsieh Y.-S. (2012). Effects of maternal confidence and competence on maternal parenting stress in newborn care. J. Adv. Nurs..

[10] Moon R., Fu L. (2022). Infant death syndrome and the committee on fetus and newborn. Sleep-Related Infant Deaths: Updated 2022 Recommendations for Reducing Infant Deaths in the Sleepview. Pediatrics.

[11] Nadège J., Loret de Mola C., Gary J., Mesenburg M., Freitas da Silveira M. (2019). Prenatal and postnatal maternal depression and infant hospitalization and mortality in the first year of life: A systematic review and meta-analysis. J. Affect. Disord..

[12] Tomori C. (2022). Overcoming barriers to breastfeeding. Best Pract. Res. Clin. Obstet. Gynaecol..

[13] Cruise S., O’Reilly D. (2014). The influence of parents, older siblings, and non-parental care on infant development at nine months of age. Infant Behav. Dev..

[14] De Sousa Machado T., Chur-Hansen A., Due C. (2020). First-time mothers’ perceptions of social support: Recommendations for best practice. Health Psychol. Open.

[15] Foley S., Hughes C. (2018). Great expectations? Do mothers’ and fathers’ prenatal thoughts and feelings about the infant predict parent-infant interaction quality? A meta-analytic review. Dev. Rev..

[16] Younglee C., Hye Y. (2021). Developing and evaluating a mobile-based parental education program for preventing unintentional injuries in early childhood: A randomized controlled trial. Asian Nurs. Res..

